# Oncogenic microRNA-181d binding to OGT contributes to resistance of ovarian cancer cells to cisplatin

**DOI:** 10.1038/s41420-021-00715-6

**Published:** 2021-12-08

**Authors:** Wei Huang, Ling Chen, Kean Zhu, Donglian Wang

**Affiliations:** 1 Department of Gynaecology, Hunan Provincial People’s Hospital, (The First Affiliated Hospital of Hunan Normal University), Changsha, 410000 P. R. China; 2 Department of Gynaecology, Hunan Provincial Maternal and Child Health Care Hospital, Changsha, 410008 P. R. China

**Keywords:** Cancer, Diseases

## Abstract

Ovarian cancer (OC), a common gynecological cancer, is characterized by a high malignant potential. MicroRNAs (miRNAs or miRs) have been associated with the chemo- or radiotherapeutic resistance of human malignancies. Herein, the current study set out to explore the regulatory mechanism of miR-181d involved in the cisplatin (DDP) resistance of OC cells. Firstly, in-situ hybridization method was performed to identify miR-181d expression in ovarian tissues of DDP-resistant or DDP-sensitive patients. In addition, miR-181d expression in A2780 cells and A2780/DDP cell lines was determined by RT-qPCR. Gain- and loss-of-function experiments were then performed to characterize the effect of miR-181d on OC cell behaviors. We probed the miR-181d affinity to OGT, as well as the downstream glycosylation of KEAP1 and ubiquitination of NRF2. Further, in vivo experiments were performed to define the role of miR-181d in tumor resistance to DDP. miR-181d was highly expressed in the ovarian tissues of DDP-resistant patients and the A2780/DDP cell line. Ectopic expression of miR-181d augmented DDP resistance in OC cells. In addition, miR-181d was found to target the 3′UTR of OGT mRNA, and negatively regulate the OGT expression. Mechanistic results indicated that OGT repressed NRF2 expression through glycosylation of KEAP1, thereby inhibiting the DDP resistance of OC cells. Furthermore, miR-181d negatively orchestrated the OGT/KEAP1/NRF2 axis to enhance the OC resistance to DDP in vivo. Overall, these findings suggest that miR-181d-mediated OGT inhibition restricts the glycosylation of KEAP1, and then reduces the ubiquitination and degradation of NRF2, leading to DDP resistance of OC. This study provides new insights for prevention and control of OC.

## Introduction

Ovarian cancer (OC) is one of the most fatal malignancies affecting women, with limited targeted treatment options and high incidence of metastasis [1]. A large proportion of mortality associated with OC is attributed to detection at a later and highly untreatable stage, and the occurrence of resistance to chemotherapy [2]. Cisplatin (DDP) is a first-line chemotherapeutic agent for the treatment of OC on the basis of platinum [3]. It has been noted that accurate prognostic strategies with the assistance of molecular targets would aid in the prognosis of OC patients [4].

Emerging evidence has identified the regulatory effect of microRNAs (miRNAs or miRs) on malignant phenotypes of tumor cells, which may function as promising therapeutic targets in the treatment of OC [5]. More notably, the abnormal upregulation of miR-181d has been documented in multiple cancers, such as pancreatic cancer, gastric cancer, and colorectal cancer [6–8]. Besides, the oncogenic role of miR-181d has also been elaborated in gastric cancer, wherein miR-181d elevation was found to be indicative of unsatisfactory prognoses and tumor aggressiveness [7]. Owing to the various evidences of miR-181d participation in numerous malignancies, exploration of the role of miR-181d in OC could prove effective in terms of treatment strategies.

Initial bioinformatics analysis in the current study also revealed the presence of putative binding sites of miR-181d on the 3′untranslated region (UTR) of O-linked N-acetylglucosamine (GlcNAc) transferase (OGT). Intriguingly, evidence reported by Zhou et al. highlighted the inhibitory impact of OGT in the resistance of OC cells to DDP, which was realized through repressing SNARE complex formation and DDP-induced autophagy [9]. Furthermore, the transcriptional effects of OGT were also previously associated with inactivation of nuclear factor E2-related factors, like nuclear factor E2-related factor 2 (NRF2), in various expression datasets of cancers, wherein kelch-like ECH-associated protein 1 (KEAP1) inversely mediated the NRF2 protein, as a direct substrate of OGT [10]. In addition, NRF2 overexpression, through stimulating antioxidant potential, has also been uncovered to bring about a significant elevation in *cis*-diamminechloroplatinum resistance of OC cells [11]. Moreover, the KEAP1/NRF2 pathway is involved in the chemoresistance in OC through governing oxidative damage in OC cells [12]. As a result, we performed this study to test our hypothesis that the miR-181d/OGT/KEAP1/NRF2 axis plays a regulatory role in the progression of OC.

## Results

### miR-181d was highly expressed in DDP-resistant OC tissues or cells

**Fig. 1:**
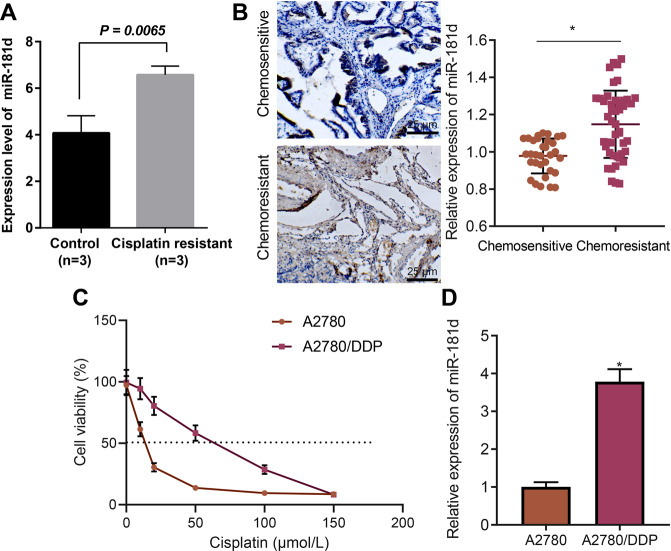
miR-181d is highly expressed in DDP-resistant OC tissues or cells. **A** Expression of miR-181d in DDP-resistant and DDP-sensitive OC cells in the GSE148251 dataset. **B** In-situ hybridization assay of the miR-181d expression in clinical tissue samples of chemo-resistant (*n* = 46) and chemo-sensitive (*n* = 32) OC patients. **C** CCK-8 assay evaluation of viability of A2780 and A2780/DDP cells after treatment with DDP at different concentrations (0, 10, 20, 50, 100, and 150 μmol/L) for 48 h. IC50 of A2780 = 12.72 μmol/L, and IC50 of A2780 DDP = 55.03 μmol/L. **D** RT-qPCR detection of miR-181d expression in A2780/DDP and A2780 cells, normalized to U6. Cell experiments were repeated three times independently. *p < 0.05 versus A2780 cells or chemo-sensitive OC patients.

Firstly, analysis on the GSE148251 dataset indicated that the levels of miR-181d were much higher in DDP-resistant OC cells than that in DDP-sensitive OC cells (Fig. 1A). Next, the in-situ hybridization assay indicated that the expression of miR-181d in OC tissues of patients with chemo-resistance was found to be higher than those in chemo-sensitive patients (Fig. 1B). It is worth noting that no abnormal expression of the miR-181 family members occurred in the clinical samples of OC patients (Supplementary Fig. 1A).

Furthermore, isogenic DDP-sensitive and DDP-resistant OC cell lines A2780 and A2780/DDP were incorporated in the current study. The IC50 value of A2780/DDP cells was found to be nearly four times higher than that of A2780 cells, as observed using CCK-8 assay (Fig. 1C). As a result, DDP concentration in the treated cells was set to 50 μmol/L in subsequent experiments. Additionally, miR-181d expression was higher in the A2780/DDP cell line compared to that in the A2780 cell line (Fig. 1D). Furthermore, the levels of miR-181d were detected to be increased in COC1/DDP, SKOV3/DDP, and OVCAR3/DDP cells compared with that in the corresponding parental cells, which was consistent with the data in A2780 cells (Supplementary Fig. 1B). Literature has reported that miR-199a, miR-136, miR-98, and miR-506 can reduce the sensitivity of OC cells to DDP by promoting the repair of DDP-induced DNA damage [13, 14]. No abnormal expression was observed in their expression in A2780/DDP, COC1/DDP, SKOV3/DDP, and OVCAR3/DDP cells (Supplementary Fig. 1C). In summary, miR-181d was highly expressed in DDP-resistant OC cells, and was consequently selected for further experimentation.

### miR-181d promoted DDP resistance in OC cells

**Fig. 2:**
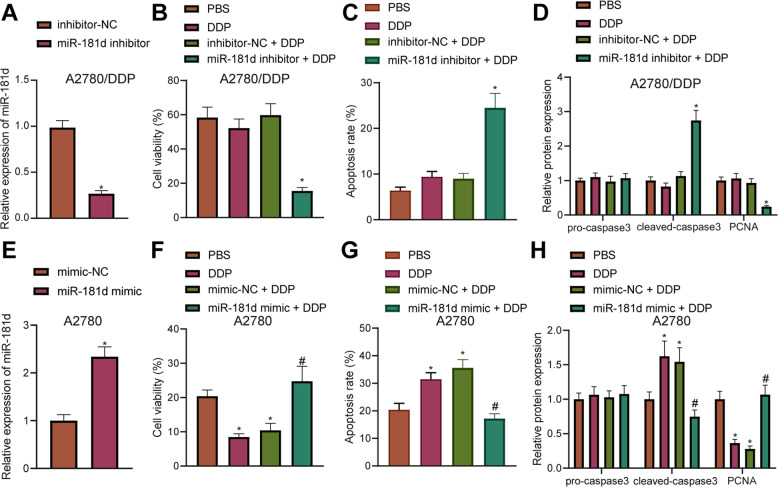
miR-181d promotes resistance of OC cells to DDP. **A** RT-qPCR detection of miR-181d expression in A2780/DDP cells after miR-181d inhibitor treatment normalized to U6. **B** CCK-8 assay of viability of A2780/DDP cells after miR-181d inhibitor treatment. **C** Flow cytometric analysis of A2780/DDP cell apoptosis after miR-181d inhibitor treatment in the presence or absence of DDP treatment. **D** Western blot assay of apoptosis-related proteins pro-caspase3, cleaved caspase-3, and proliferation-related protein PCNA in A2780/DDP cells normalized to β-actin after miR-181d inhibitor treatment. **E** RT-qPCR detection of miR-181d expression in A2780 cells after miR-181d mimic treatment normalized to U6. **F** CCK-8 assay of viability of A2780 cells after miR-181d mimic treatment. **G** Flow cytometric analysis of A2780 cell apoptosis after miR-181d mimic treatment in the presence or absence of DDP treatment. **H** Western blot assay of apoptosis-related proteins pro-caspase3, cleaved caspase-3, and proliferation-related protein PCNA in A2780 cells after miR-181d mimic treatment. Cell experiments were repeated three times independently. *p < 0.05 versus A2780/DDP cells treated with inhibitor-NC or A2780 cells treated with mimic-NC.

To further investigate the effect of miR-181d on chemotherapy sensitivity of DDP-resistant OC cells, A2780 and A2780/DDP cells were treated with miR-181d mimic and miR-181d inhibitor, respectively. According to the previous results in Fig. 1C, the concentration of DDP treatment was 50 μmol/L. miR-181d expression was diminished in A2780/DDP cells following treatment with miR-181d inhibitor (Fig. 2A), while being elevated in A2780 cells after miR-181d mimic treatment (Fig. 2E). In addition, miR-181d inhibitor reduced the viability of A2780/DDP cells after DDP treatment (Fig. 2B), while miR-181d mimic treatment augmented the viability of A2780 cells after DDP treatment (Fig. 2F). Meanwhile, flow cytometric detection results indicated that miR-181d inhibitor treatment brought about increased apoptosis of A2780/DDP cells with or without DDP treatment (Fig. 2C), while inhibition of other miR-181 family members in A2780/DDP cells did not generate similar effects (Supplementary Fig. 2A, B). miR-181d mimic treatment reduced A2780 cell apoptosis in the presence or absence of DDP treatment (Fig. 2G). In addition, Western blot assay results revealed that miR-181d inhibitor treatment elevated the expression of cleaved caspase-3 and diminished those of proliferating cell nuclear antigen (PCNA, one of the widely recognized proliferation markers conferring an important role in DNA replication and the initiation of cell proliferation [15, 16] in A2780/DDP cells, while miR-181d mimic treatment brought about the opposite trends in A2780 cells following DDP treatment (Fig. 2D, H, Supplementary Fig. 3A, B). However, overexpression of other miR-181 family members in A278 cells did not generate similar effects (Supplementary Fig. 2A, B). These findings demonstrated that miR-181d enhanced the resistance of OC cells to DDP.

### OGT is a direct target of miR-181d

**Fig. 3:**
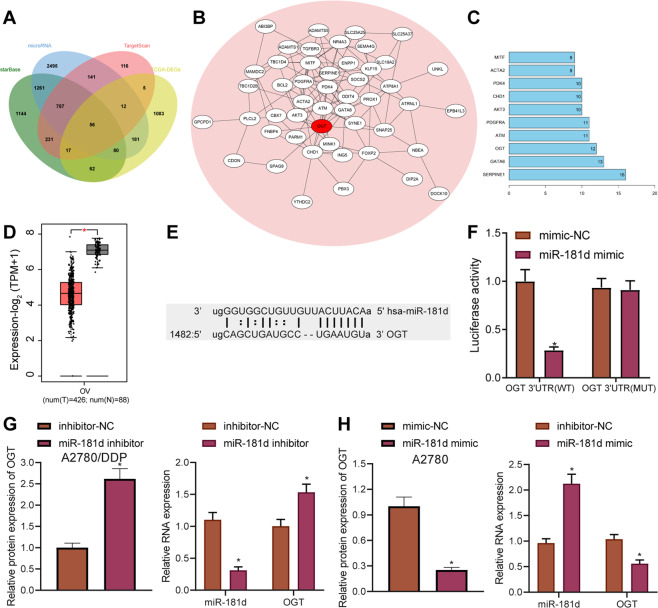
OGT is a direct target gene of miR-181d. **A** Venn diagram of the target genes of miR-181d predicted by the starBase, microRNA.org, and TargetScan databases. The four ellipses in the figure represent the significantly downregulated genes in OC samples retrieved from the GEPIA and the candidate target genes predicted by the three databases. The middle part represents the intersection of the four sets of data. **B** Candidate target gene interaction network diagram. Each ellipse in the figure represents a gene, the lines between the ellipses indicate the presence of interaction between genes, and the red represents OGT. **C** The degree value statistics of core genes in the gene interaction network diagram. The more interacting genes represent the higher degree value and higher core degree. The *x*-axis in the figure represents the degree value, and the *y*-axis represents the gene. **D** Differential expression of OGT gene in OC and normal samples in the TCGA and GTEx databases. The *x*-axis indicates the sample type, the *y*-axis indicates the expression, the red box plot indicates the tumor sample, and the gray box plot indicates the normal sample (T Tumor, N Normal, *p < 0.01 versus normal samples). **E** Putative miR-181d binding sites in the OGT 3′UTR predicted by the microRNA.org database. **F** Binding of miR-181d to OGT confirmed by dual luciferase reporter assay. **G** Western blot assay and RT-qPCR detection of OGT protein and mRNA expression in A2780/DDP cells after miR-181d inhibitor treatment. **H** Western blot assay of OGT protein in A2780 cells after miR-181d mimic treatment normalized to β-actin. Cell experiments were repeated three times independently. *p < 0.05 versus A2780/DDP cells treated with inhibitor-NC or A2780 cells treated with mimic-NC or HEK293T co-transfected with mimic-NC and OGT 3′UTR WT.

This study further explored the molecular mechanism of miR-181d and its impact on DDP sensitivity. The target genes of miR-181d were predicted using the starBase database, microRNA.org database, and TargetScan, and the results were subsequently intersected with the significantly downregulated genes in OC obtained through TCGA, which reared a total of 56 candidate target genes (Fig. 3A). A gene interaction network diagram was plotted for these 56 candidate target genes and the degree value of core genes was counted, which identified that OGT was at the core position in the gene network diagram (Fig. 3B, C). In addition, OGT was indicated to be significantly downregulated in OC according to TCGA and GTEx (Fig. 3D). Immunohistochemistry analysis showed higher expression of OGT in clinical samples of chemo-sensitive OC patients than that in those of chemo-resistant OC patients (Supplementary Fig. 4A).

Prior evidence reported by Zhou et al. has also highlighted the inhibitory impact of OGT on resistance of OC cells to DDP [8]. As a result, the microRNA.org database was retrieved, which predicted the binding site between miR-181d and OGT (Fig. 3E). Additionally, dual luciferase reporter assay results manifested that miR-181d mimic inhibited the luciferase activity of OGT 3′UTR WT, while exerting no significant effects on that of OGT 3′UTR MUT (Fig. 3F). Moreover, miR-181d inhibitor increased OGT protein and mRNA expression (Fig. 3G, Supplementary Fig. 3C). Meantime, miR-181d mimic led to a reduction in OGT expression (Fig. 3H, Supplementary Fig. 3D). Homology analysis of the miR-181 family members revealed that miR-181a-d was highly conserved at the 5′end. However, there was still a slight difference between the 3′end and the target region of OGT (Supplementary Fig. 4B). In addition, the decline in expression of OGT induced by miR-181d was found to be the most significant in A2780/DDP cells (Supplementary Fig. 4C). In conclusion, miR-181d can specifically target OGT, and negatively regulate its expression in OC cells.

### miR-181d promoted DDP resistance in OC cells by downregulating OGT

**Fig. 4:**
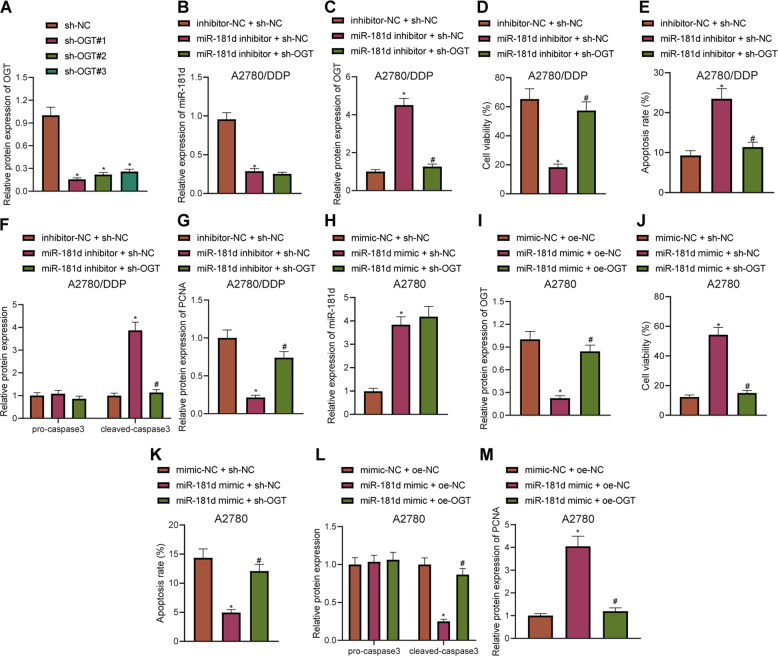
miR-181d promotes resistance of OC cells to DDP by downregulating OGT. **A** Western blot assay of sh-OGT knockdown efficiency in A2780/DDP cells. A2780/DDP cells were treated with inhibitor-NC + sh-NC, miR-181d inhibitor + sh-NC, or miR-181d inhibitor + sh-OGT. **B** RT-qPCR detection of miR-181d expression in A2780/DDP cells normalized to U6. **C** Western blot assay of OGT expression in A2780/DDP cells normalized to β-actin. **D** CCK-8 assay of A2780/DDP cell viability. **E** Flow cytometric detection of A2780/DDP cell apoptosis. **F** Western blot assay of pro-caspase3 and cleaved caspase-3 expression and quantitative analysis in A2780/DDP cells normalized to β-actin. **G** Western blot assay of PCNA expression and quantitative analysis in A2780/DDP cells normalized to β-actin. A2780 cells were treated with mimic-NC + oe-NC, miR-181d mimic + oe-NC, or miR-181d mimic + oe-OGT. **H** RT-qPCR detection of miR-181d expression in A2780 cells normalized to U6. **I** Western blot assay of OGT expression in A2780 cells normalized to β-actin. **J** CCK-8 assay of A2780 cell viability. **K** Flow cytometric detection of A2780 cell apoptosis. **L** Western blot assay of pro-caspase3 and cleaved caspase-3 expression and quantitative analysis in A2780 cells normalized to β-actin. **M** Western blot assay of PCNA expression and quantitative analysis in A2780 cells normalized to β-actin. Cell experiments were repeated three times independently. *p < 0.05 versus A2780/DDP cells treated with sh-NC, or inhibitor-NC + sh-NC or A2780 cells treated with mimic-NC + oe-NC. #*p* < 0.05 versus A2780/DDP cells treated with miR-181d inhibitor + sh-NC or A2780 cells treated with miR-181d mimic + oe-NC.

Our initial results indicated that miR-181d negatively regulated OGT, which led us to speculate whether this targeting relationship affected the DDP resistance of OC cells. Subsequently, A2780/DDP cells were treated with miR-181d inhibitor and sh-OGT. Western blot assay confirmed that sh-OGT#1 exhibited the highest silencing efficiency, so sh-OGT#1 was selected for further experiments (Fig. 4A, Supplementary Fig. 5A). In addition, miR-181d inhibitor was found to reduce the miR-181d expression and increased OGT expression. Moreover, in the presence of miR-181d inhibitor, silencing of OGT did not alter the miR-181d expression, but reduced the OGT expression in A2780/DDP cells (Fig. 4B, C, Supplementary Fig. 5B), indicating that the treatments in each group were successful. Meanwhile, CCK-8 assay results demonstrated that miR-181d inhibitor treatment reduced the viability of A2780/DDP cells, while cell viability was restored after the co-treatment of miR-181d inhibitor and sh-OGT (Fig. 4D). Flow cytometric detection showed that following treatment with miR-181d inhibitor, A2780/DDP cell apoptosis was increased, while co-treatment with miR-181d inhibitor and sh-OGT led to reduced cell apoptosis (Fig. 4E). Western blot assay further revealed that miR-181d inhibitor treatment brought about increased expression of cleaved caspase-3 in A2780/DDP cells, while co-treatment of miR-181d inhibition and OGT silencing reduced the cleaved caspase-3 expression (Fig. 4F, Supplementary Fig. 5C). Additionally, the expression patterns of proliferation-related protein PCNA were detected using Western blot assay, and it was found that miR-181d inhibitor treatment caused an increase in PCNA expression in A2780/DDP cells, while co-treatment of miR-181d inhibitor and sh-OGT restored the PCNA expression (Fig. 4G, Supplementary Fig. 5D).

Additionally, A2780 cells were treated with miR-181d mimic and oe-OGT, and it was found that miR-181d mimic treatment in A2780 cells increased the expression of miR-181d and diminished OGT expression. In the presence of miR-181d mimic, oe-OGT brought about no change in miR-181d expression and increase in OGT expression in A2780 cells (Fig. 4H, I, Supplementary Fig. 5E), indicating that the treatments in each group were successful. For A2780 cells treated with DDP, cell viability was observed to be augmented after miR-181d mimic treatment, while being suppressed following co-treatment with miR-181d mimic and oe-OGT (Fig. 4J). Flow cytometric detection demonstrated that apoptosis was decreased in the A2780 cell line treated with DDP after miR-181d mimic treatment, and increased after miR-181d mimic + oe-OGT treatment (Fig. 4K). Western blot assay results revealed that in the A2780 cell line treated with DDP, the expression of cleaved caspase-3 was diminished following miR-181d mimic treatment, while being restored after co-treatment with miR-181d mimic and oe-OGT (Fig. 4L, Supplementary Fig. 5F). Also, miR-181d mimic treatment in A2780 cells increased the expression of PCNA, while miR-181d mimic and oe-OGT co-treatment brought about the opposite effect (Fig. 4M, Supplementary Fig. 5G). The above results indicated that miR-181d augments DDP resistance in OC cells by downregulating OGT.

### OGT promotes glycosylation of KEAP1, and increases its stability and expression, thus enhancing the ubiquitination and degradation of NRF2 and inhibiting DDP resistance of OC cells

**Fig. 5:**
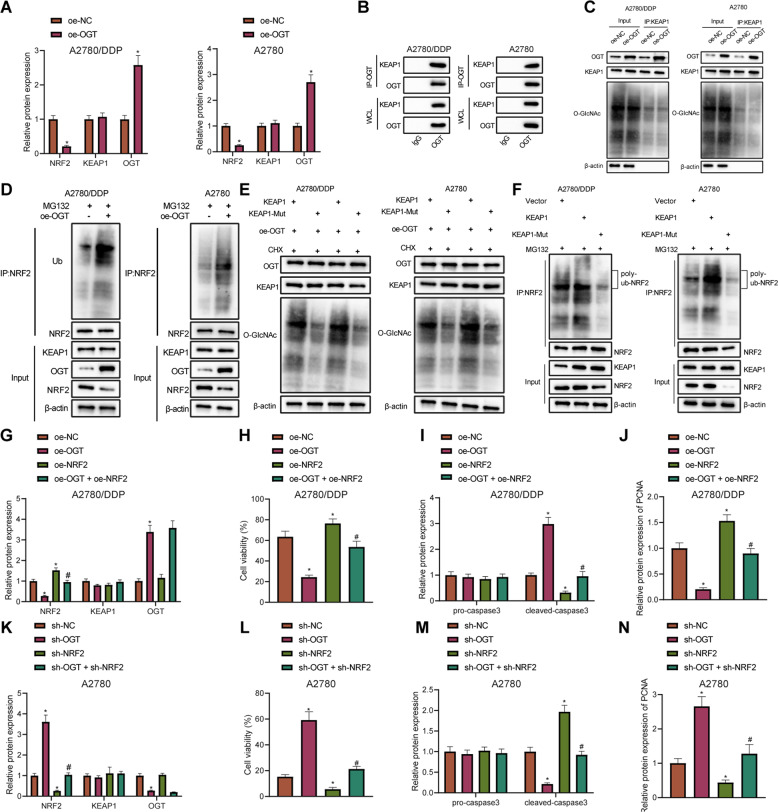
OGT augments glycosylation of KEAP1, and increases its stability and expression, consequently promoting the ubiquitination and degradation of NRF2, and thereby inhibiting DDP resistance of OC cells. **A** Western blot assay of the expression and quantitative analysis of KEAP1 in A2780 and A2780/DDP cells overexpressing OGT normalized to β-actin. **B** Co-IP assay of the interaction between OGT and KEAP1 in A2780 cells or A2780/DDP cells. **C** Co-IP assay of KEAP1 glycosylation in A2780 cells or A2780/DDP cells. **D** Co-IP assay of the ubiquitination modification level of NRF2 in A2780 cells or A2780/DDP cells. **E** Western blot assay of changes in KEAP1 glycosylation in A2780 cells or A2780/DDP cells. **F** Co-IP assay of the ubiquitination degree of NRF2 in A2780 cells or A2780/DDP cells. A2780/DDP cells were treated with oe-NC + sh-NC, oe-OGT, oe-OGT + sh-KEAP1 or oe-OGT + oe-NRF2. **G** Western blot assay of the expression and quantitative analysis of OGT, KEAP1, and NRF2 in A2780/DDP cells normalized to β-actin. **H** CCK-8 assay of the change of A2780/DDP cell viability. **I** Western blot assay of cleaved caspase-3 expression and quantitative analysis in A2780/DDP cells normalized to β-actin. **J** Western blot assay of PCNA expression and quantitative analysis in A2780/DDP cells normalized to β-actin. A2780 cells were treated with oe-NC + sh-NC, sh-OGT, sh-OGT + oe-KEAP1 or sh-OGT + sh-NRF2. **K** Western blot assay determination of KEAP1, OGT, and NRF2 expression and quantitative analysis in A2780 cells normalized to β-actin. **L** CCK-8 assay assessment of A2780 cell viability. **M** Western blot assay of the cleaved caspase-3 expression and quantitative analysis in A2780 cells normalized to β-actin. **N** Western blot assay of PCNA expression and quantitative analysis in A2780 cells normalized to β-actin. Cell experiments were repeated three times independently. *p < 0.05 versus A2780/DDP or A2780 cells treated with oe-NC, A2780/DDP or A2780 cells treated with sh-NC or A2780/DDP or A2780 cells treated with oe-NC + sh-NC. #*p* < 0.05 versus A2780/DDP cells treated with oe-OGT or A2780 cells treated with sh-OGT.

A previous study has shown that OGT can induce O-glycosylation of KEAP1 at S104, which in turn promotes the ubiquitination and degradation of NRF2, and high levels of NRF2 are also related to the cisplatin resistance of OC cells [10, 17]. Thus, we speculate that OGT can regulate KEAP1/NRF2 through glycosylation in ovarian cancer cells to regulate the cisplatin resistance of OC cells. Results of Western blot assay demonstrated that after overexpressing OGT in A2780 cells and A2780/DDP cell lines, KEAP1 expression remained unchanged, while NRF2 expression was diminished (Fig. 5A, Supplementary Fig. 6A, B). In addition, Co-IP assay results verified that OGT interacted with KEAP1 in A2780 cells and A2780/DDP cell lines (Fig. 5B). Moreover, OGT overexpression in A2780 cells and A2780/DDP cell lines was found to increase the degree of glycosylation of KEAP1 (Fig. 5C), accompanied by an elevation in NRF2 ubiquitination (Fig. 5D). Furthermore, after mutating the KEAP1 S104 glycosylation site (KEAP1 MUT), the degree of KEAP1 glycosylation and NRF2 ubiquitination in the KEAP1 MUT group were noted to be reduced compared to the KEAP1 WT group (Fig. 5E, F).

Subsequently, after oe-OGT treatment, OGT expression was increased and NRF2 expression was diminished in the A2780/DDP cells, while in the presence of oe-OGT, further silencing of NRF2 brought about a reduction in the KEAP1 expression, and further treatment of oe-NRF2 induced a potent rise in the NRF2 expression (Fig. 5G, Supplementary Fig. 6C). CCK-8 assay and Western blot assay after DDP treatment also demonstrated that A2780/DDP cell viability was reduced following oe-OGT treatment, accompanied by reduced PCNA expression and increased cleaved caspase-3 expression. These trends could be reversed by treatment with oe-OGT + sh-KEAP1 or oe-OGT + oe-NRF2 (Fig. 5H–J, Supplementary Fig. 6D, E).

Next, it was confirmed that OGT expression was reduced in the A2780 cells silencing OGT, and NRF2 expression were increased; OGT expression was diminished while the KEAP1 expression was augmented in the A2780 cells treated with sh-OGT + oe-KEAP1; OGT expression was reduced, but KEAP1 and NRF2 expression remained unchanged in A2780 cells treated with sh-OGT + sh-NRF2 (Fig. 5K, Supplementary Fig. 6F). Moreover, the results of CCK-8 assay and Western blot assay revealed that A2780 cell viability and PCNA expression were increased, while cleaved caspase-3 expression was diminished in DDP-treated A2780 cells after OGT silencing, but KEAP1 overexpression or NRF2 silencing reversed these trends (Fig. 5L–N, Supplementary Fig. 6G, H). The above results suggested that OGT glycosylation stabilized KEAP1 to suppress the expression of NRF2, thereby inhibiting DDP resistance in OC cells.

### miR-181d regulated DDP resistance in OC cells through OGT/KEAP1/NRF2 axis

Finally, we aimed to verify whether miR-181d regulates drug resistance in OC cells through the OGT/KEAP1/NRF2 axis. Co-IP results demonstrated that inhibition of miR-181d in A2780/DDP cells promoted the glycosylation of KEAP1 while promoting the ubiquitination of NRF2, which was reversed by further knockdown of OGT (Supplementary Fig. 7A, B). The above data indicated that miR-181d targeted OGT and repressed its expression, thereby inhibiting the glycosylation of KEAP1 and reducing the ubiquitination of NRF2.

**Fig. 6:**
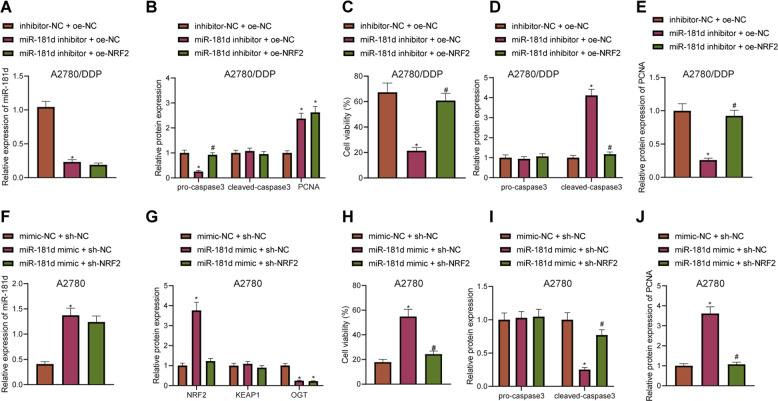
miR-181d regulates DDP resistance of OC cells through the OGT/KEAP1/NRF2 axis. A2780/DDP cells were treated with inhibitor-NC + oe-NC, miR-181d inhibitor + oe-NC or miR-181d inhibitor + oe-NRF2. **A** RT-qPCR detection of miR-181d expression in A2780/DDP cells normalized to U6. **B** Western blot assay of OGT, KEAP1, and NRF2 expression and quantitative analysis in A2780/DDP cells normalized to β-actin. **C** CCK-8 assay of A2780/DDP cell viability. **D** Western blot assay of pro-caspase3 and cleaved caspase-3 expression and quantitative analysis in A2780/DDP cells normalized to β-actin. **E** Western blot assay of PCNA expression and quantitative analysis in A2780/DDP cells normalized to β-actin. A2780 cells were transfected with mimic-NC + sh-NC, miR-181d mimic + sh-NC or miR-181d mimic + sh-NRF2. **F** RT-qPCR detection of miR-181d expression in A2780 cells normalized to U6. **G** Western blot assay of the expression and quantitative analysis of OGT, KEAP1, and NRF2 in A2780 cells normalized to β-actin. **H** CCK-8 assay of A2780 cell viability. **I** Western blot assay of the pro-caspase3 and cleaved caspase-3 expression and quantitative analysis in A2780 cells normalized to β-actin. **J** Western blot assay of PCNA expression and quantitative analysis in A2780 cells normalized to β-actin. Cell experiments were repeated three times independently. *p < 0.05 versus A2780/DDP cells treated with inhibitor-NC + oe-NC or A2780 cells treated with mimic-NC + sh-NC. #*p* < 0.05 versus A2780 cells treated with miR-181d mimic + sh-NC or A2780/DDP cells treated with miR-181d inhibitor + oe-NC.

After treatment with miR-181d inhibitor or co-treatment with miR-181d inhibitor and oe-NRF2, miR-181d expression was found to be reduced (Fig. 6A). Meanwhile, Western blot assay demonstrated that after treatment with miR-181d inhibitor, OGT expression was increased, KEAP1 expression remained unchanged, and NRF2 expression was diminished. After co-treatment with miR-181d inhibitor and oe-NRF2, OGT expression was increased, and KEAP1 and NRF2 expression remained unchanged (Fig. 6B, Supplementary Fig. 8A). Analysis of the DDP-treated A2780/DDP cell line by the CCK-8 assay and Western blot assay revealed that viability of A2780/DDP cells and PCNA expression were diminished, accompanied by a potent increase in cleaved caspase-3 expression following treatment with miR-181d inhibitor, which were abrogated after co-treatment with miR-181d inhibitor and oe-NRF2 (Fig. 6C–E, Supplementary Fig. 8B, C).

In addition, the levels of miR-181d were decreased in COC1/DDP cells treated with miR-181d inhibitor or combined with oe-NRF2 (Supplementary Fig. 9A). Western blot assay results displayed an increase in the expression of OGT, a decline in the expression of NRF2, and no changes in the expression of KEAP1 in COC1/DDP cells upon treatment with miR-181d inhibitor. Meanwhile, in response to the combined treatment with miR-181d inhibitor and oe-NRF2, the expression of OGT was elevated while that of KEAP1 remained unchanged, and NRF2 expression did not change significantly in COC1/DDP cells (Supplementary Fig. 9B). Furthermore, overexpression of NRF2 in COC1/DDP cells could also reverse the decrease in cell viability and increase in cell apoptosis caused by miR-181d inhibition (Supplementary Fig. 9C, D).

Moreover, miR-181d mimic alone or miR-181d mimic + sh-NRF2 brought about an elevation in miR-181d expression in A2780 cells (Fig. 6F). Meanwhile, miR-181d mimic alone elevated the OGT expression, while miR-181d mimic + sh-NRF2 treatment led to diminished NRF2 expression when compared to miR-181d mimic alone in A2780 cells (Fig. 6G, Supplementary Fig. 8D). CCK-8 assay and Western blot assay after DDP treatment showed that A2780 cell viability and PCNA expression were increased, accompanied by reduced cleaved caspase-3 expression after miR-181d mimic treatment, whereas co-treatment with miR-181d mimic and sh-NRF2 reversed these trends (Fig. 6H–J, Supplementary Fig. 8E, F). These findings indicated that miR-181d modulates DDP resistance in OC cells through regulation of the OGT/KEAP1/NRF2 axis.

### miR-181d regulates the OGT/KEAP1/NRF2 axis to promote OC resistance to DDP in vivo

**Fig. 7:**
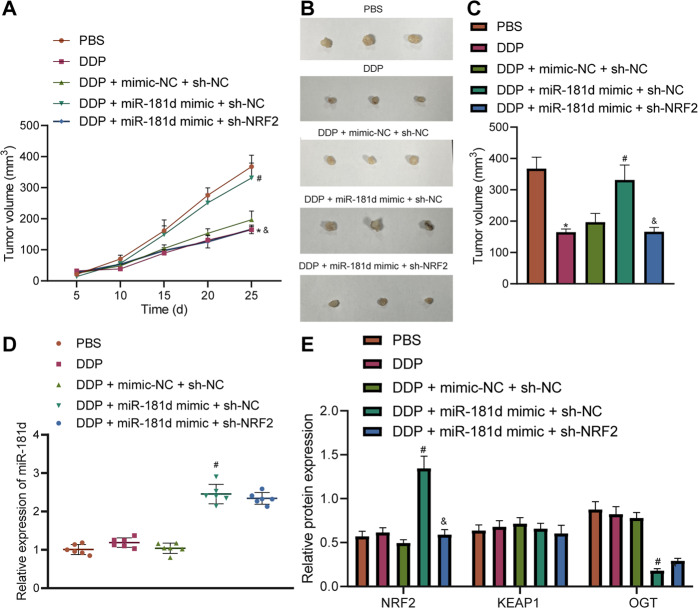
miR-181d regulates the OGT/KEAP1/NRF2 axis to promote OC Resistance to DDP in vivo. A2780 cells stably transfected with mimic-NC + sh-NC, miR-181d mimic + sh-NC or miR-181d mimic + sh-NRF2 and untreated A2780 cells were xenografted subcutaneously into the DDP-treated nude mice. **A** Tumor volume of nude mice measured at 5, 10, 15, 25 days after cell injection. **B** Representative images showing xenografts in nude mice. **C** Quantitative analysis of panel **B**. **D** RT-qPCR detection of miR-181d expression in tumor tissues of nude mice normalized to U6. **E** Western blot assay of the expression and quantitative analysis of NRF2, KEAP1, and OGT proteins in nude mouse tumor tissues normalized to β-actin. *p < 0.05 versus nude mice treated with PBS. #*p* < 0.05 versus nude mice treated with DDP + mimic-NC + sh-NC. & *p* < 0.05 versus nude mice treated with DDP + miR-181d mimic + sh-NC. *n* = 6 for nude mice in each group.

In order to confirm the physiological function of miR-181d/OGT/KEAP1/NRF2, A2780 cell-implanted xenograft tumor models were established to clarify the effect of OGT on tumor formation in mice. DDP treatment inhibited tumor growth while further miR-181d mimic treatment led to opposite results. Combined treatment with miR-181d mimic and sh-NRF2 reduced tumor growth (Fig. 7A–C). In addition, the results of RT-qPCR and Western blot assay suggested that the expression of miR-181d, OGT, and NRF2 remained un-altered in mouse tumor tissues. Besides, miR-181d mimic increased the expression of miR-181d and NRF2 but reduced that of OGT, while in the presence of DDP + miR-181d mimic + sh-NRF2, only NRF2 expression was diminished (Fig. 7D, E). The above results implied that miR-181d promoted OC resistance to DDP through regulation of the OGT/KEAP1/NRF2 axis.

## Discussion

**Fig. 8:**
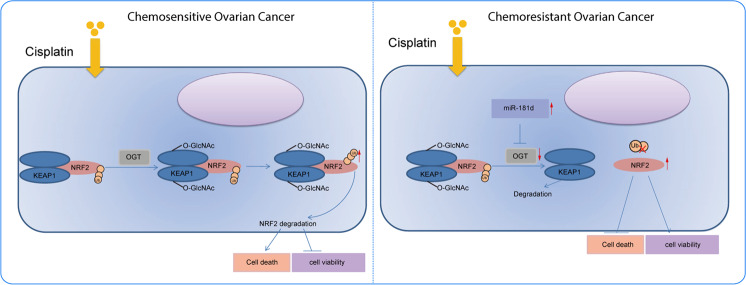
Schematic diagram of the mechanism by which miR-181d affects OC chemoresistance via the OGT/KEAP1/NRF2 axis. miR-181d directly targets OGT and inhibits the expression of OGT, thereby inhibiting the glycosylation of KEAP1, reducing the ubiquitination, and degradation of NRF2 and promoting the expression of NRF2. By this mechanism, the viability of OC cells is promoted while cell apoptosis is suppressed, ultimately inducing the chemoresistance of OC.

OC is a leading cause of death in relation to gynecological tumors, with 70–80% patients affected by relapse after chemotherapy and chemoresistance serving as the most significant cause of mortality [18]. Nevertheless, the hard-done work of our peers has highlighted that miRNAs have the potential to serve as novel therapeutic targets for OC [19]. Our work indicated that miR-181d overexpression augmented DDP resistance of OC cells through the OGT/KEAP1/NRF2 axis (Fig. 8).

Data of our work revealed that miR-181d was highly expressed in DDP-resistant OC tissues and the A2780 cell line. Moreover, Wang et al. also highlighted the tumor-supporting role of miR-181d in breast cancer through both in vitro and in vivo explorations [20]. Prior reports have suggested the importance of miRNAs in DDP resistance; for example, miR-199a elevates the expression of ABCG2 and thereby augments the resistance of colorectal cancer stem cells to DDP [21]. In addition, deficiency of miR-136, miR-9 and miR-506 can reduce the sensitivity of OC cells to DDP by promoting the repair of DDP-induced DNA damage [22–24]. The expression of the above-mentioned miRNAs in drug-resistant OC cells (A2780/DDP, COC1/DDP, SKOV3/DDP, and OVCAR3/DDP cells) showed no abnormal expression in these cells, which indicated that maybe the DDP sensitivity regulation involved in these miRNAs is a cell line-specific regulation. In the current study, findings indicated that miR-181d promoted DDP resistance in OC cells as evidenced by augmented cell viability and diminished cell apoptosis. Meanwhile, Zhuo et al. also illustrated the high expression patterns and promotive role of miR-181d in hepatocellular carcinoma cells resistant to DDP, which is very much in line with our findings [25].

Furthermore, we proceeded to probe the specific downstream mechanistic actions of miR-181d in OC, which subsequently revealed that OGT was a direct target of miR-181d, and miR-181d inversely mediated the OGT expression. Similarly, studies have suggested that the oncogenic roles of the miR-181 family in breast cancer cells is achieved through targeting PHLPP2 and INPP4B phosphatases, thus accelerating cancer cell cycle progression and proliferative potential [26]. In view of the binding affinity of miR-181d to OGT confirmed by dual luciferase reporter assay, it would be reasonable to speculate that the tumor-supporting properties of miR-181d are associated with OGT inhibition. Additional experimentation in the current study yielded evidence indicating that when the OGT expression was diminished by ectopic miR-181d, the DDP-resistant OC cells were consequently induced. Prior studies have also documented downregulated expression of OGT in DDP-resistant OC tissues and cells, wherein inhibition of OGT led to DDP resistance through modulation of SNAP-29 [9].

Not stopping there, we further unmasked the mechanism behind the DDP resistance induced by miR-181d-mediated OGT inhibition in OC. OGT augmented the glycosylation of KEAP1 and stabilized the KEAP1 protein, consequently leading to promotion of NRF2 ubiquitination and degradation, thereby increasing the sensitivity of OC cells to DDP. A previous study has shown that OGT induces O-glycosylation of KEAP1 at S104, which elevates the ubiquitination and degradation of NRF2, which is in accordance with our findings [10]. In another study focusing on DDP resistance in OC cells, oxidative stress and antioxidant genes were taken into serious consideration, which were found to be involved with the p62-mediated KEAP1/NRF2/ARE pathway [27]. Furthermore, highly expressed NRF2 was also previously correlated with DDP resistance in OC cells [17], while knockdown of NRF2 was observed to impede DDP resistance in OC cells [28]. Conclusively, our study has presented functional experiments demonstrating that miR-181d regulated the OGT/KEAP1/NRF2 axis to promote the DDP resistance in vitro and tumor growth in vivo of OC.

The current study provides evidence indicating that miR-181d binds to OGT and augments DDP resistance in OC cells through the KEAP1/NRF2 axis. Our findings support the stance that the miR-181d/OGT/KEAP1/NRF2 axis could potentially shed new light on the understanding of chemoresistance in OC for the development of personalized therapies. In addition, miR-181d can be used as a novel biological marker for predicting the chemotherapy sensitivity of patients with OC or a drug target in the treatment of OC. However, our research is still at the preclinical stage, and the investigation on the mechanism of action of the miR-181d-dependent OGT/KEAP1/NRF2 axis is not yet well-elucidated. Thus, it is also recommended in future studies to extend the regulator network into other markers.

## Materials/subjects and methods

### Study subjects

Paraffin-embedded tumor tissues were collected from 78 patients diagnosed with OC at the Hunan Provincial People’s Hospital (The First Affiliated Hospital of Hunan Normal University) and Hunan Provincial Maternal and Child Healthcare Hospital from June 2006 to June 2019. All patients underwent initial surgery and subsequently completed DDP-based chemotherapy. Clinical information of 78 patients with OC is described in Supplementary Table 1. The patients were classified into a chemo-resistant group (*n* = 32) and a chemo-sensitive group (*n* = 46). The chemoresistance was defined as follows: during the course of postoperative chemotherapy, the disease continued to develop; after 6 courses of chemotherapy, CA125 did not fall into the normal range; or patients who relapsed within 6 months after the primary chemotherapy. The chemosensitivity was defined as follows: the patients relapsed after more than 6 months or did not relapse.

### In-situ hybridization

Paraffin-embedded slices were de-paraffinized and subjected to proteinase K (Roche, Shanghai, China) treatment. Next, 300 ng/mL miR-181d probe (Sigma) was added for 16-h hybridization at 65 °C. Sections were then incubated with anti-digoxygenin-AP (AP-conjugated Fab fragments) (Roche, #11093 274 910) diluted at 1: 2000 for 1 h at 25 °C. Subsequently, color reaction was conducted in BM Purple (Roche, # 1 442 074 001) in the dark. Finally, the sections were photographed and ImageProPlus software was applied for grayscale analysis.

### Cell culture, transfection, and grouping

Human embryonic kidney (HEK293T) cells (American Type Culture Collection, Manassas, VA, USA; CRL-1573) were cultured in DMEM; (Solarbio, Beijing, China) with 10% FBS in a humidified incubator at 37 °C with 5% CO_2_.

The OC cell line A2780 and DDP-resistant cell line A2780/DDP were purchased from Shanghai Meixuan Biological Science and Technology Ltd. (Shanghai, China). OC cell lines COC1 and SKOV3 as well as their DDP-resistant cell lines COC1/DDP and SKOV3/DDP were purchased from Wuhan Procell Life Science & Technology Co., Ltd. (Wuhan, China). Parental OVCAR3 cell line and the DDP-resistant cell line OVCAR3/DDP were purchased from Shanghai Chuanqiu Biotechnology Co., Ltd. (Shanghai, China). First, 0.5 μg/mL DDP was added to the drug-resistant cell culture medium to maintain drug resistance. The culture medium was renewed with DDP-free medium three days before the experiment. All cells were cultured in RPMI-1640 medium (Hyclone, Logan, UT, SH30809.01) encompassing 10% FBS (Zhejiang Tianhang Biotechnology Co., Ltd., Zhejiang, China) and penicillin/streptomycin (final concentration = 100 IE, Sigma, St. Louis, MO) at 37 °C with 5% CO_2_.

The logarithmically growing cells were seeded in 6-well plates at a density of 3 × 10^5^ cells/well. When the cell confluence reached 70–80%, transfection was performed in the light of the manuals of Lipofectamine 3000 kits (L3000008, Invitrogen, Carlsbad, CA). Briefly, each centrifuge tube was added with 250 μL serum-free DMEM-1640, incubated with 4 μg of corresponding plasmids (synthesized by GenePharma, Shanghai, China), and then diluted with Lipofectamine 3000 at room temperature for 20 min to make the plasmids fully encapsulated by liposomes. Next, the mixture of plasmids was incubated with A2780 or A2780/DDP cells in a 6-well plate. A2780/DDP cells were then transfected with miR-181d inhibitor, sh-OGT, oe-OGT, oe-NRF2, inhibitor-NC, oe-NC, sh-NC alone or in combination. Meanwhile, A2780 cells were transfected with miR-181d mimic, oe-OGT, sh-OGT, oe-KEAP1, sh-NRF2, mimic-NC, oe-NC, sh-NC alone or in combination. miR-181d mimic, miR-181d inhibitor, mimic-NC, and inhibitor-NC were purchased from Shanghai GenePharma Co., Ltd. (Shanghai, China). The abovementioned silencing plasmids pGPU6 (C02001) were procured from GenePharma (Shanghai, China), while the overexpression plasmids pcDNA3.1 (V79520) were procured from Thermo Fisher Scientific Inc. (Waltham, MA). The full-length sequence of OGT with KpnI and XhoI restriction sites was artificially synthesized, then ligated to pcDNA3.1 using T4 ligase and introduced into competent E. coli. Incubation was conducted for 24 h at 37 °C for amplification and plasmid extraction. After sequencing and identification, the eukaryotic expression vector was constructed.

Following experiments were conducted 24 h after transfection. For DDP-treated cells, different concentrations of DDP (0, 10, 20, 50, 100, and 150 μmo/L, Qilu Pharmaceutical Co., Ltd., Jinan, China) were dissolved in phosphate buffer saline (PBS) to treat the cells, and the cells were harvested after 48 h for subsequent experimentation.

### CCK-8 assay

The viability of the cells was evaluated as per the protocols of CCK-8 kits (Dojindo Laboratories, Tokyo, Japan). Briefly, the cells were seeded in a 96-well plate at a density of 1 × 10^3^ cells/well (100 μL/well). Next, 10 μL of CCK-8 solution was supplemented to each well. Half an hour later, the absorbance value at 450 nm was examined using a 680-microplate reader (Bio-Rad, Hercules, CA).

### Flow cytometry

The cell apoptosis was examined as per the protocols of Annexin V-FITC/PI kit (BD Biosciences Pharmingen, San Jose, CA). The cells (200 μL) were centrifuged and resuspended in 100 μL binding buffer, followed by addition with 2 μL Annexin V-FITC (20 μg/mL; V13241; Life Technologies, Carlsbad, CA). Afterwards, each sample was supplemented with 1 μL PI (50 μg/mL). The flow cytometric detection was performed within 30 min.

### Dual luciferase reporter assay

The synthetic OGT 3′UTR gene fragment was inserted into the pGL3-control (Promega, Madison, WI) using endonuclease sites, XhoI and BamHI. The mutation site of the complementary sequence of the seed sequence was designed on the OGT wild type (WT). The mutant sites were found in the cancer somatic mutation catalog (https://cancer.sanger.ac.uk/cosmic) (KEAP1 mutants: GCTTATCTTCTGGTACCCCATGCA). Following restriction endonuclease cleavage, the target fragment was inserted into the pGL3-control vector using T4 DNA ligase. The correctly sequenced luciferase reporter plasmids were then co-transfected into the HEK293T cells with miR-181d mimic or mimic NC. Forty-eight hours later, luciferase activity was assayed on a Luminometer TD-20/20 detector (E5311, Promega) using Dual-Luciferase Reporter Assay System kits (Promega).

### Co-IP assay

The interaction between endogenous OGT and KEAP1 protein was assessed using Co-IP assay. Pierce IP buffer containing protease and phosphatase inhibitors was utilized to lyse the A2780 or A2780/DDP cells. Cell lysates were then probed overnight at 4 °C with the rabbit antibodies to KEAP1 (4678, 1:50, Cell Signaling Technology, Danvers, MA), NRF2 (ab137550, 1: 100, Abcam, Cambridge, UK), and IgG (IgG; ab7099, 1: 100, Abcam). Cells were subsequently added with protein G beads (Dynabeads, Thermo Fisher Scientific), and slowly vortexed at 4 °C for 8 h to couple the antibody and protein with the protein A + G agarose beads. After the immunoprecipitation reaction, the cells were centrifuged to obtain agarose beads, which were washed 3-4 times with 1 mL of lysis buffer. Finally, Western blot assay was applied to examine the expression of KEAP1.

### Ubiquitination assay

For the endogenous NRF2-Ub assay, A2780 and A2780/DDP cells were transfected with KEAP1 wild-type (WT) and KEAP1 mutant (MUT) for 48 h. After 6-h treatment with 10 μM MG132 (a protease inhibitor, HY-13259, MedChemExpress, Monmouth Junction, NJ), the cells were lysed in 1% SDS RIPA buffer. The sonicated cells were subjected to Co-IP assay, with rabbit anti-rat NRF2 antibody (137550, 1: 100, Abcam) incubated with the diluted cell lysate (last 0.1% SDS) at 4 °C overnight. Following another incubation with protein G beads at 4 °C for 8 h, the cells were washed three times in IP buffer. Finally, the degree of NRF2 ubiquitination was examined using Western blot assay.

### Glycosylation detection

A2780 cells or A2780/DDP cells were treated with 25 μg/mL cycloheximide (CHX) for 1 h to block translation and then with 50 μM 5SGlcNAc for additional 10 h. Whole-cell lysates (WCLs) were analyzed for NRF2 protein and global O-GlcNAcylation by Western blot assay.

### RT-qPCR

Total RNA content was extracted from the tissues and cells using TRIzol reagents (16096020, Thermo Fisher Scientific). For mRNA detection, the extracted RNA was reversely transcribed into cDNA using Reverse Transcription Kit (M1701, Promega). The cDNA sample was incubated at 80 °C for 5 min to inactivate the reverse transcriptase for subsequent RT-qPCR detection. For miRNA detection, the extracted RNA was reversely transcribed into cDNA using miRNA First Strand cDNA Synthesis (Tailing Reaction) Kit (B532451, Shanghai Sangon Biotechnology Co., Ltd., Shanghai, China). Downstream primers were provided by the kit. RT-qPCR was performed using TaqMan MicroRNA Assay and TaqMan^®^ Universal PCR Master Mix. GAPDH was regarded as an internal reference of mRNAs while U6 as an internal reference of miRNA. Three replicate wells were set for each sample. The primer sequences are shown in Supplementary Table 2. Relative quantitative method (2^-△△CT^ method) was used to calculate the relative transcription level of the tested gene [29].

### Western blot assay

Total protein content was extracted from the tissues and cells using RIPA lysis buffer (R0010, Solarbio), and the protein concentration was determined with a bicinchoninic acid protein assay kit (Guangzhou Jiebeisi Biotechnology Co., Ltd., Guangzhou, China). Proteins were subsequently loaded (40 µg per sample) and separated with gel electrophoresis using 6, 10, and 15% SDS-polyacrylamide gel electrophoresis. After the separated protein was transferred onto a polyvinylidene fluoride membrane (Millipore, Billerica, MA), the membrane was blocked with Tris-buffered saline with Tween 20 solution encompassing 5% bovine serum albumin at room temperature. The membrane was then probed at 4 °C overnight with diluted primary antibodies (1: 1000; Abcam) to rabbit anti-OGT (ab177941), rabbit anti-KEAP1 (ab196346), rabbit anti-NRF2 (ab137550), rabbit anti-Ubiquitin (ab7780), mouse anti-O-GlcNAc (ab2739), rabbit anti-cleaved caspase-3 (ab2302), and caspase-3 (ab13847), rabbit anti-PCNA (ab32552), and rabbit anti-β-actin (ab179467). Afterwards, the membrane was incubated with secondary antibody goat anti-rabbit IgG antibody (ab97051, 1: 2000, Abcam) or goat anti-mouse IgG antibody (ab205719, 1: 2000, Abcam) at room temperature. The bands were visualized using ECL reagent, and band exposure imaging was performed on an Image Quant LAS 4000 C gel imager (GE Healthcare, Pittsburgh, PA).

### Xenograft tumor in nude mice

Twenty-four five-week-old female BALB/c nude mice (Vital River Laboratory Animal Technology Co., Ltd., Beijing, China) were raised under specific pathogen-free (SPF) conditions. These mice were grouped into four groups, with six mice in each group. The stably transfected A2780 cells were resuspended in sterile PBS. The A2780 cells were untreated or stably transfected with mimic-NC + sh-NC, miR-181d mimic + sh-NC, or miR-181d mimic + sh-NRF2. A total of 2 × 10^6^ cells in 100 µL PBS were inoculated subcutaneously into the ventral side of nude mice. Cells were assigned into PBS and DDP groups. On the 5^th^ day, 10^th^ day, 15^th^ day, 20^th^ day, and 25^th^ day of cell inoculation, the volume of subcutaneous tumors in mice was measured. Tumor volume = width × length^2^ × 0.5. DDP treatment was conducted 5 days after cell injection. Each mouse was injected intraperitoneally with DDP (3 mg/kg) or PBS every two days until the end of the experiment. The mice were then euthanized and tumors were obtained for further analyses.

### Microarray-based gene expression profiling

OC-related expression dataset GSE148251 [17] was retrieved from the Gene Expression Omnibus (GEO) database. GSE148251 dataset contained 3 DDP-sensitive OC cells and 3 DDP-resistant OC cells. With DDP-sensitive OC cells regarded as the control, differential analysis was conducted using R language “limma” package with |logFC | > 2 and *p* < 0.05 set as the threshold to screen differentially expressed miRNAs. The starBase database, microRNA.org database, and TargetScan database were adopted to predict the target genes of miR-181d. In addition, the Gene Expression Profiling Interactive Analysis 2 database was adopted to retrieve the significantly downregulated genes in the OC samples included in The Cancer Genome Atlas (TCGA), and the expression patterns of OGT in OC and normal samples included in TCGA and Genotype-Tissue Expression (GTEx) were also obtained. The interaction of candidate target genes was analyzed using the STRING database, and a gene interaction network diagram was plotted using the Cytoscape v3.7.1 software, followed by calculation of degree value.

### Statistical analysis

Statistical analyses were performed using the SPSS 22.0 software (IBM Corp. Armonk, NY). Measurement data were summarized as mean ± standard deviation. Independent-sample *t* test was used for two-group data comparison. One-way analysis of variance (ANOVA) was carried out for data comparison among multiple groups followed by Tukey’s post-hoc test. Two-way ANOVA was employed to determine cell viability under different concentrations, while repeated measures ANOVA with Bonferroni post-hoc test was performed for data comparison at different time points. A value of *p* < 0.05 was considered statistically significant.

### Abbreviations

UTR untranslated region, OC Ovarian cancer, miRNAs or miRs microRNAs, DDP Cisplatin, OGT O-linked N-acetylglucosamine (GlcNAc) transferase, NRF2 nuclear factor E2-related factor 2, KEAP1 kelch-like ECH-associated protein 1, RPMI Roswell Park Memorial Institute, oe overexpression, sh short hairpin, DMEM Dulbecco’s modified Eagle’s medium, FBS fetal bovine serum, NC negative control, CCK Cell counting kit, PBS phosphate-buffered saline, MUT mutant type, WT wild type, RT-qPCR Reverse transcription quantitative polymerase chain reaction, cDNA complementary DNA, RIPA radio-immunoprecipitation assay, SDS sodium dodecyl sulfate, SPF specific pathogen free, Co-IP Co-immunoprecipitation, PCNA proliferating cell nuclear antigen, GEO Gene Expression Omnibus, TCGA The Cancer Genome Atlas, GTEx Genotype-Tissue Expression, ANOVA analysis of variance.

## Supplementary information

Supplementary Figure Legends

Supplementary Figure 1

Supplementary Figure 2

Supplementary Figure 3

Supplementary Figure 4

Supplementary Figure 5

Supplementary Figure 6

Supplementary Figure 7

Supplementary Figure 8

Supplementary Figure 9

Supplementary Tables

## Data Availability

The datasets generated/analyzed during the current study are available from the corresponding author upon reasonable request.
