# Supplementary material for: Oncogenic microRNA-181d binding to OGT contributes to resistance of ovarian cancer cells to cisplatin
Source: Cell Death Discov. 2021 Dec 8;7:379. doi: 10.1038/s41420-021-00715-6 (PMC8651739; doi:10.1038/s41420-021-00715-6)
Supplement: Supplementary file 1 — Supplementary Figure Legends [file 41420_2021_715_MOESM1_ESM.docx]

**Supplementary Figure Legends**

**Supplementary Fig. 1 Expression of relevant miRNAs.** A, Expression of the miR-181 family members in the clinical samples of OC patients detected by *in-situ* hybridization assay. B, Expression of miR-181d in COC1/DDP, SKOV3/DDP and OVCAR3/DDP cells along with the corresponding parental cells detected by RT-qPCR. * *p* < 0.05 versus corresponding parental cells. C, Expression of miR-136, miR-9 and miR-506 in A2780/DDP, COC1/DDP, SKOV3/DDP and OVCAR3/DDP cells along with the corresponding parental cells detected by RT-qPCR. The cell experiment was run in triplicate independently.

**Supplementary Fig. 2 Other members of the miR-181 family do not affect the sensitivity of OC cells to DDP.** A, Flow cytometric analysis of apoptosis of A2780 and A2780/DDP cells treated with miR-181a mimic, miR-181b mimic, miR-181c mimic or miR-181d mimic. B, CCK-8 analysis of viability of A2780 and A2780/DDP cells treated with miR-181a mimic, miR-181b mimic, miR-181c mimic or miR-181d mimic. * *p* < 0.05 versus A2780 and A2780/DDP cells treated with mimic NC or inhibitor NC. The cell experiment was run in triplicate independently.

**Supplementary Fig. 3 Representative images of Western blots for densitometric quantification of figure 2D (A), 2H (B), 3G (C), and 3H (D).** The cell experiment was run in triplicate independently.

**Supplementary Fig. 4 Targeting of the other members of the miR-181 family on OGT.** A, Immunohistochemistry analysis of OGT protein in clinical samples of chemo-resistant (n = 46) and chemo-sensitive (n = 32) OC patients. * *p* < 0.05 versus chemo-sensitive OC patients. B, Homology analysis of the miR-181 family members. C, Expression of OGT in A2780/DDP cells treated with miR-181a inhibitor, miR-181b inhibitor, miR-181c inhibitor or miR-181d inhibitor detected by RT-qPCR. * *p* < 0.05 versus chemo-sensitive sample or A2780/DDP cells treated with inhibitor NC. The cell experiment was run in triplicate independently.

**Supplementary Fig. 5 Representative images of Western blots for densitometric quantification of figure 4A (A), 4C (B), 4F (C), 4G (D), 4I (E), 4L (F), and 4M (G).** The cell experiment was run in triplicate independently.

**Supplementary Fig. 6 Representative images of Western blots for densitometric quantification of figure 5A (A-B), 5G (C), 5I (D), 5J (E), 5K (F), 5M (G), and 5N (H).** The cell experiment was run in triplicate independently.

**Supplementary Fig. 7 miR-181d regulates NRF2 ubiquitination through OGT.** A, Western blot assay of the glycosylation level of KEAP1 in A2780/DDP cells treated with miR-181d inhibitor, sh-OGT or the protein synthesis inhibitor CHX. B, Co-IP analysis of NRF2 ubiquitination in A2780/DDP cells treated with miR-181d inhibitor, sh-OGT or MG132. The cell experiment was run in triplicate independently.

**Supplementary Fig. 8 Representative images of Western blots for densitometric quantification of figure 6B (A), 6D (B), 6E (C), 6G (D), 6I (E), and 6J (F).**

**Supplementary Fig. 9 miR-181d regulates the resistance of COC1 OC cells to DDP through NRF2.** A, RT-qPCR detection of miR-181d expression in COC1/DDP cells treated with miR-181d inhibitor or combined with oe-NRF2. B, Western blot assay of OGT, KEAP1 and NRF2 proteins in COC1/DDP cells treated with miR-181d inhibitor or combined with oe-NRF2. C, MTT detection of COC1/DDP cell viability in response to treatment with miR-181d inhibitor or combined with oe-NRF2. D, Flow cytometric analysis of COC1/DDP cell apoptosis in response to treatment with miR-181d inhibitor or combined with oe-NRF2. * *p* < 0.05 versus COC1/DDP cells treated with inhibitor NC + oe-NC. The cell experiment was run in triplicate independently.
