# Supplementary figures and images for: Oncogenic microRNA-181d binding to OGT contributes to resistance of ovarian cancer cells to cisplatin
Source: Cell Death Discov. 2021 Dec 8;7:379. doi: 10.1038/s41420-021-00715-6 (PMC8651739; doi:10.1038/s41420-021-00715-6)

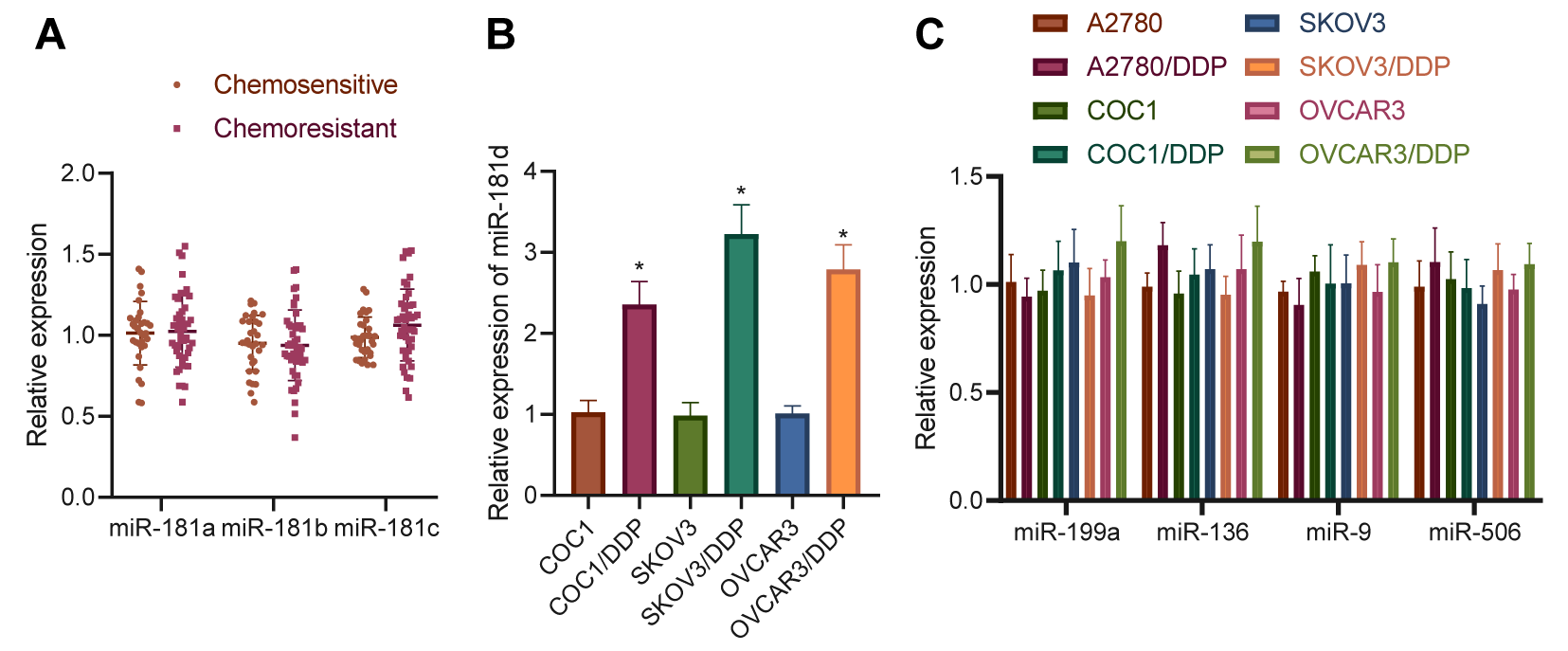

Supplement: Supplementary file 2 — Supplementary Figure 1 [file 41420_2021_715_MOESM2_ESM.tif]

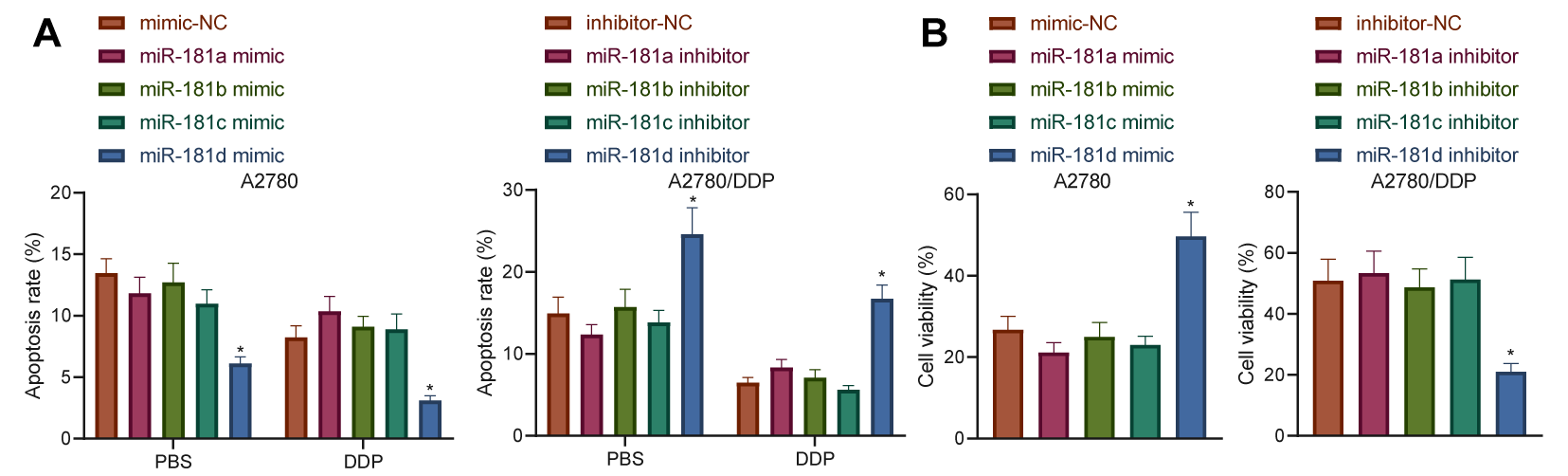

Supplement: Supplementary file 3 — Supplementary Figure 2 [file 41420_2021_715_MOESM3_ESM.tif]

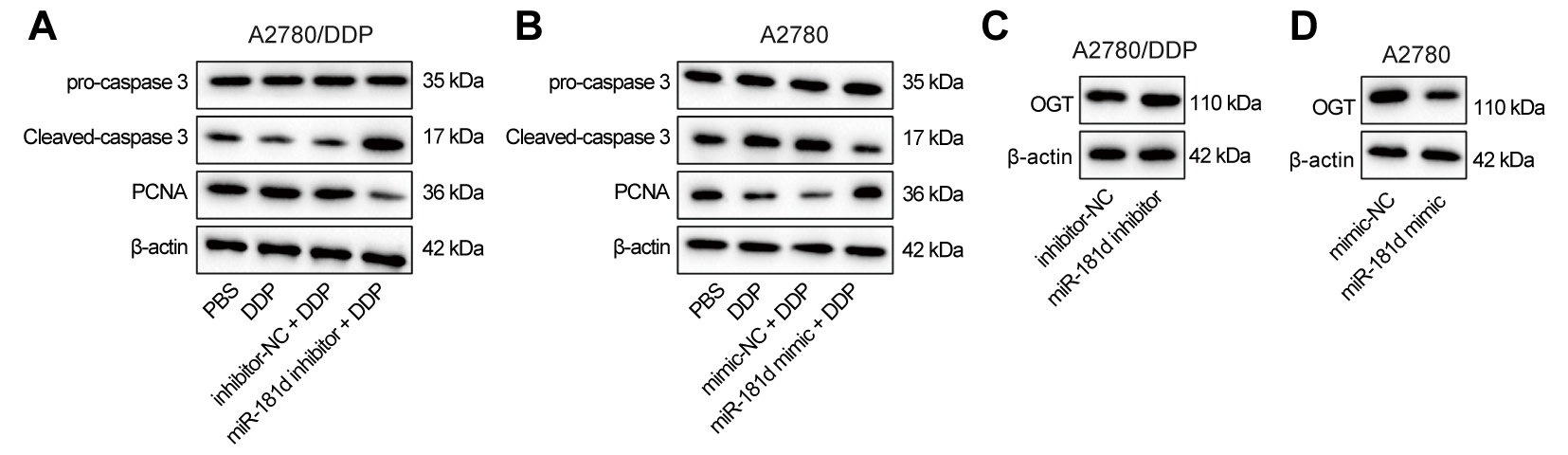

Supplement: Supplementary file 4 — Supplementary Figure 3 [file 41420_2021_715_MOESM4_ESM.tif]

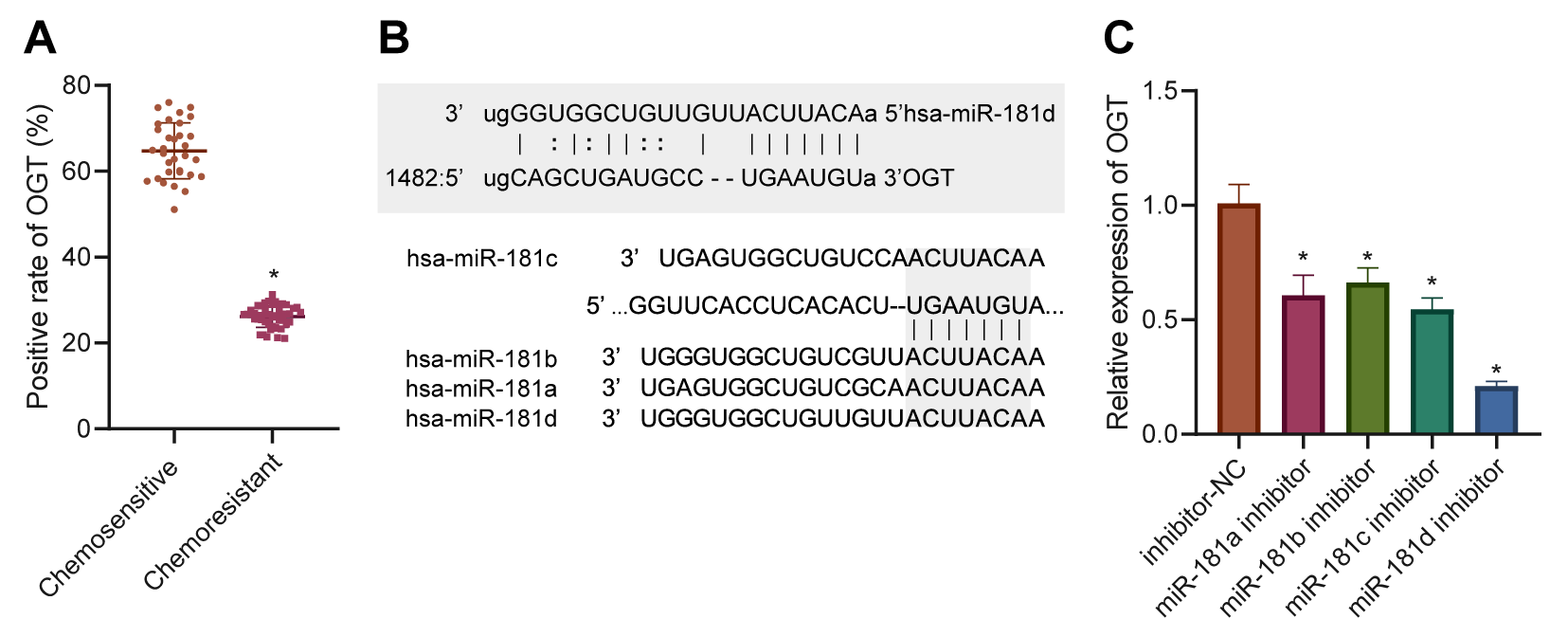

Supplement: Supplementary file 5 — Supplementary Figure 4 [file 41420_2021_715_MOESM5_ESM.tif]

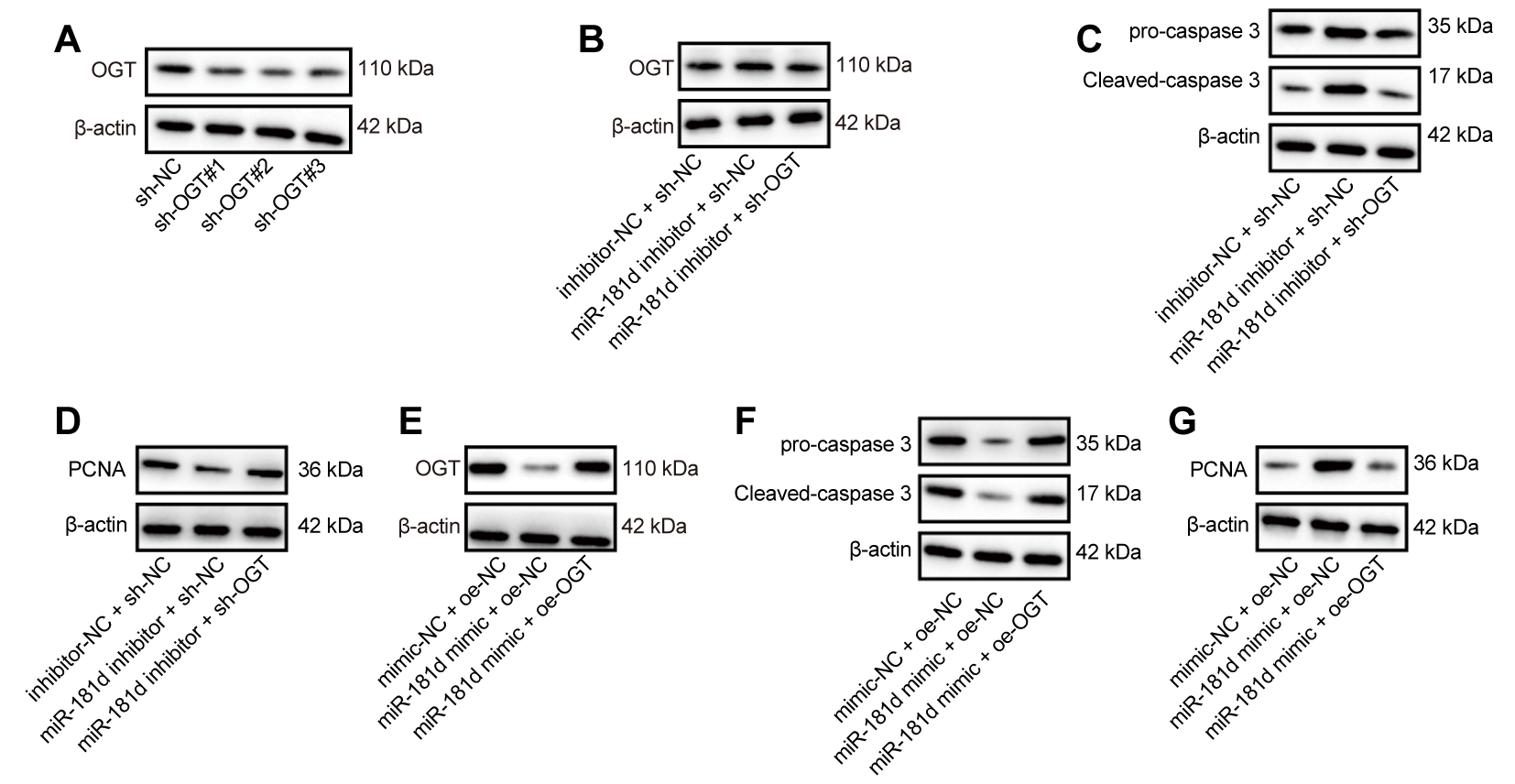

Supplement: Supplementary file 6 — Supplementary Figure 5 [file 41420_2021_715_MOESM6_ESM.tif]

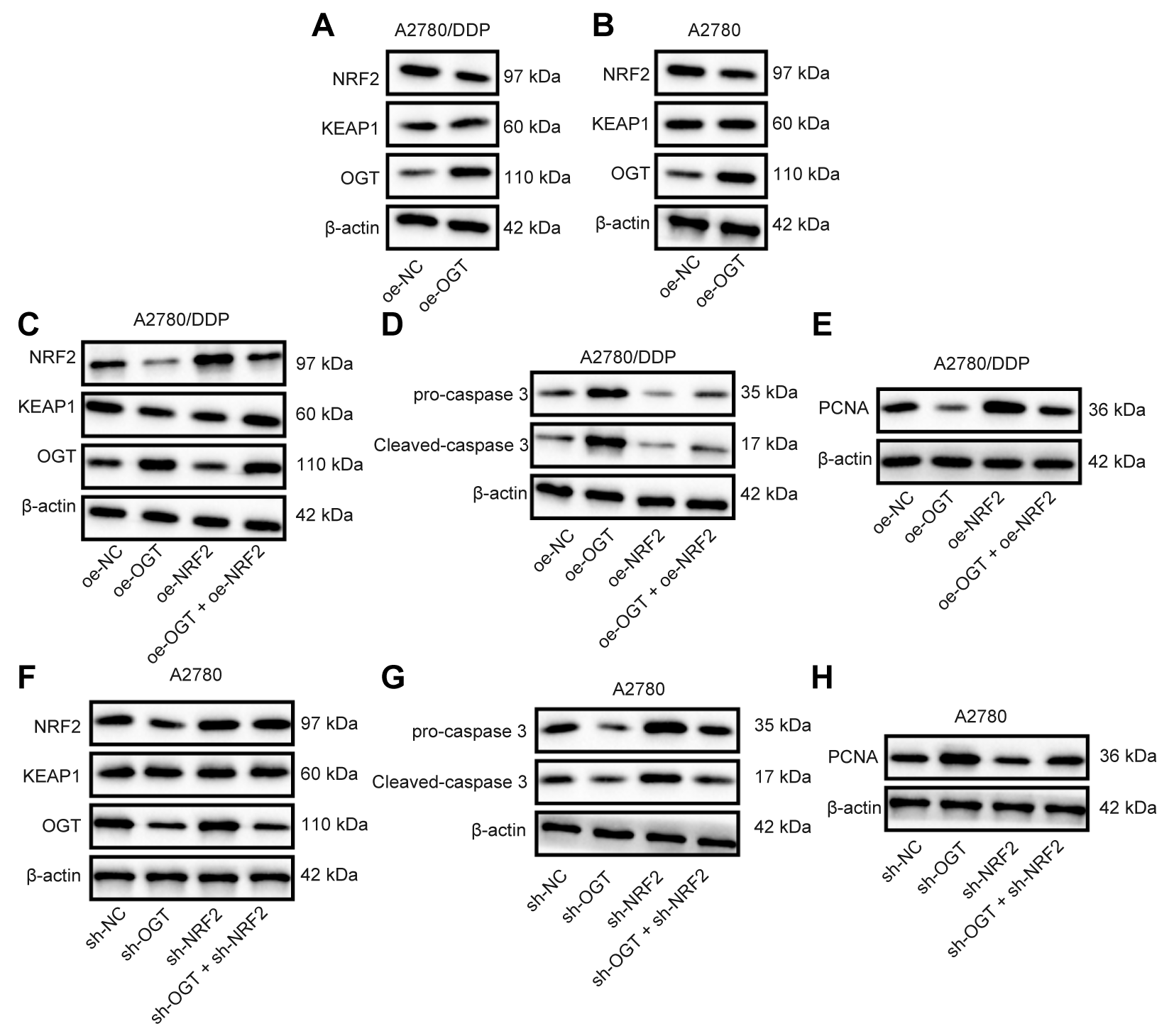

Supplement: Supplementary file 7 — Supplementary Figure 6 [file 41420_2021_715_MOESM7_ESM.tif]

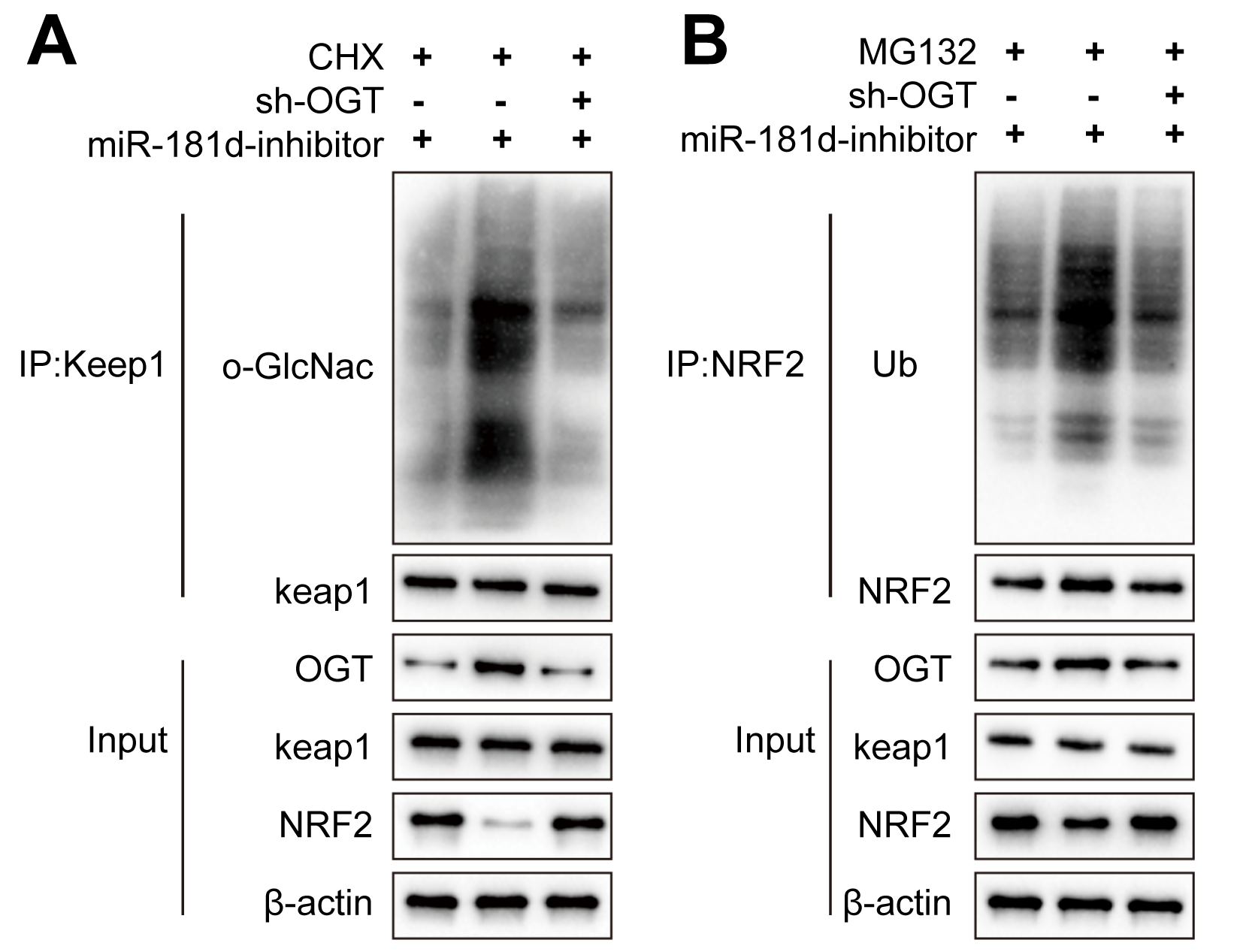

Supplement: Supplementary file 8 — Supplementary Figure 7 [file 41420_2021_715_MOESM8_ESM.tif]

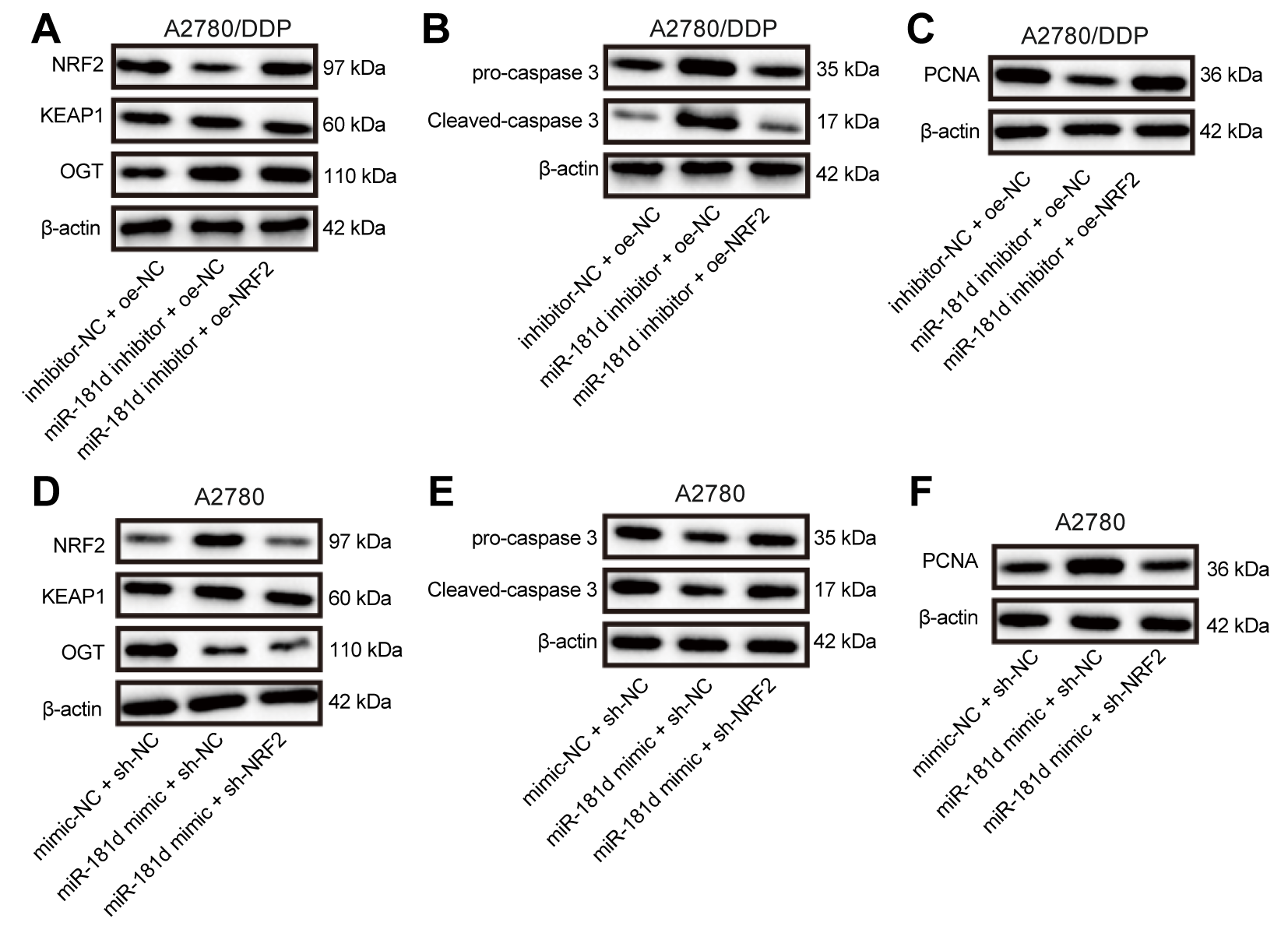

Supplement: Supplementary file 9 — Supplementary Figure 8 [file 41420_2021_715_MOESM9_ESM.tif]

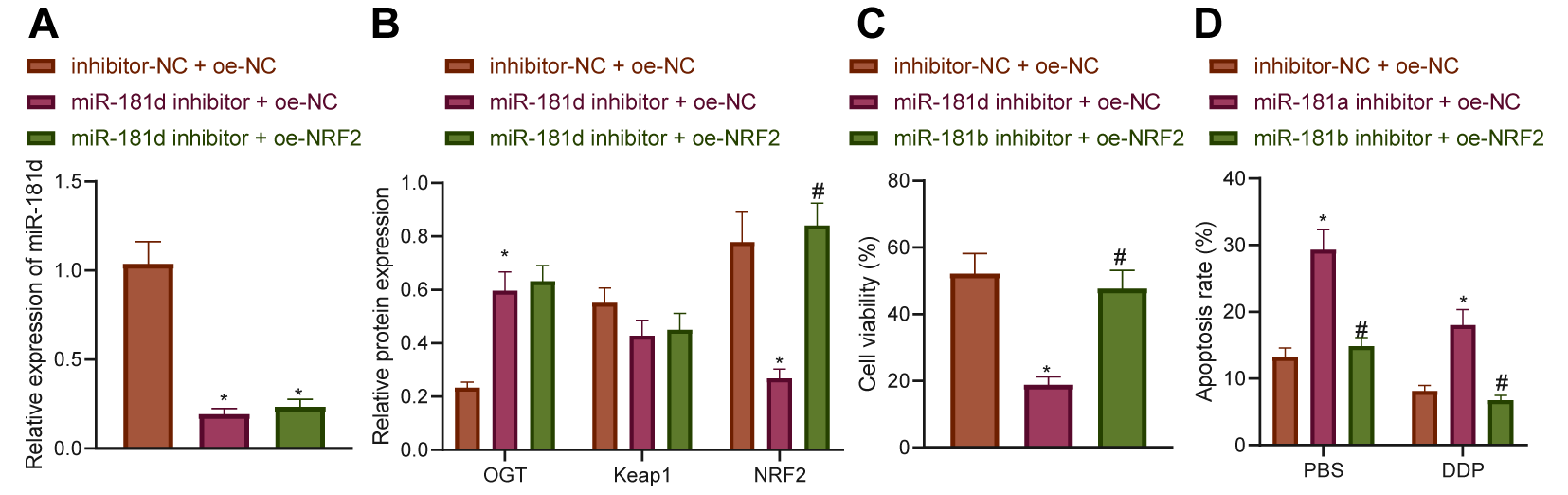

Supplement: Supplementary file 10 — Supplementary Figure 9 [file 41420_2021_715_MOESM10_ESM.tif]
