# Supplementary material for: Oncogenic microRNA-181d binding to OGT contributes to resistance of ovarian cancer cells to cisplatin
Source: Cell Death Discov. 2021 Dec 8;7:379. doi: 10.1038/s41420-021-00715-6 (PMC8651739; doi:10.1038/s41420-021-00715-6)
Supplement: Supplementary file 11 — Supplementary Tables [file 41420_2021_715_MOESM11_ESM.docx]

**Supplementary Table 1.** Clinical characteristics for 78 patients with OC

| Characteristics | Chemosensitive group  (N = 32) | Chemoresistant group  (N = 46) |
| --- | --- | --- |
| Age (years) | 51.16 ± 6.02 | 50.28 ± 5.32 |
| Clinical stage |  |  |
| Ⅰ | 18 | 16 |
| Ⅱ | 13 | 13 |
| Ⅲ | 11 | 17 |
| Pathological type |  |  |
| Serous | 26 | 37 |
| Mucinous | 6 | 9 |

**Supplementary Table 2.** Primer sequences for RT-qPCR

| Gene | Primer sequence |
| --- | --- |
| miR-181d | Forward: 5’-AACATTCATTGTTGTCGGTGGGT-3’ |
|  | Reverse: provided by the kit |
| U6 | Forward: 5’-GGGCAGGAA GAGGGCCTAT-3’ |
|  | Reverse: provided by the kit |
| OGT | Forward: 5’-CCTGGGTCGCTTGGAAGA-3’ |
|  | Reverse: 5’-CGGTTGCGTCTCAATTGCTTT-3’ |
| GAPDH | Forward: 5’-TGAACGGGAAGCTCACTGG-3’ |
|  | Reverse: 5’-TCCACCACCCTGTTGCTGTA-3’ |
